# Exploratory analyses of potential moderators of a personalized intervention to reduce preoperative anxiety: a secondary analysis of a randomized clinical trial

**DOI:** 10.1038/s41598-026-63192-w

**Published:** 2026-07-22

**Authors:** Stefan Salzmann, Frank Euteneuer, Noah Becker, Laura Kikker, Ellen Tosberg, Markus Spies, Dirk Rüsch

**Affiliations:** 1 Division of Clinical Psychology and Psychotherapy, Marburg University, Gutenbergstraße 18, 35032 Marburg, Germany; 2 Faculty of Human Sciences, Division of Clinical Psychology and Psychotherapy, Vinzenz Pallotti University, Vallendar, Germany; 3 Faculty of Human Sciences, Division of Biological Psychology and Neuroscience, Vinzenz Pallotti University, Vallendar, Germany; 4 Marburg University, Marburg, Germany; 5 Department of Anesthesia and Intensive Care, University Hospital Giessen-Marburg (Marburg Campus), Marburg, Germany

**Keywords:** Diseases, Health care, Medical research, Psychology, Psychology

## Abstract

This exploratory secondary analysis investigated whether individual patient characteristics might moderate the effectiveness of a personalized, information-based intervention aimed at reducing preoperative anxiety. The study included 122 patients scheduled for elective surgery. Anxiety was assessed repeatedly before surgery using the Amsterdam Preoperative Anxiety and Information Scale alongside numeric rating scales, capturing surgery-related anxiety, anesthesia-related anxiety, and overall anxiety. Constrained linear mixed models were applied to evaluate potential moderating effects of sociodemographic (e.g., education), psychological (e.g., neuroticism, depression, need for information, perceived physician competence and warmth), and clinical variables (e.g., smoking status, previous surgeries, type of surgery, chronic pain, premedication). The intervention consisted of a structured conversation supplemented by optional video material. The analyses provided little evidence that intervention effects systematically differed according to the examined patient characteristics. Among the examined moderators, chronic pain yielded the strongest indication of potential effect modification with regard to anesthesia-related anxiety outcomes. However, these findings did not remain statistically significant after correction for multiple testing and should therefore be considered exploratory and hypothesis-generating. Overall, the present findings provide no confirmatory evidence that the effectiveness of the intervention differs systematically across the examined patient characteristics. Future adequately powered studies are needed to determine whether specific patient characteristics influence response to personalized preoperative anxiety interventions.

**Supplementary Information:**

The online version contains supplementary material available at 10.1038/s41598-026-63192-w.

## Introduction

Preoperative anxiety is a common clinical issue, often associated with psychological distress, a desire for support^1^, and negative postoperative outcomes, such as increased pain and higher use of analgesics^2,3^. Addressing this anxiety in patients who desire support for coping with their anxiety is therefore of clinical importance. Research further indicates a considerable heterogeneity of factors associated with preoperative anxiety and a preference for personal conversations in addition to premedication to cope with preoperative anxiety^4^.

In a previous study we examined whether a brief, personalized, information-based preoperative intervention —delivered via an empathic conversation and/or a video—could reduce preoperative anxiety. The results of this study indicated that a personalized intervention significantly and clinically meaningfully reduced patients’ anxiety and also significantly lowered the desire for anxiolytic premedication offered to patients of both study groups^5^. However, it remains unclear which patients benefitted most from this intervention. There is evidence that individual patient characteristics can influence how patients benefit from interventions^6^. Therefore, it is crucial to identify relevant moderators of intervention effects to determine for whom an intervention is effective, and for whom it may even be detrimental.

In this paper, we report the results of an exploratory secondary analysis of a previously published randomized controlled trial^5^. We examined whether selected patients‘ characteristics (education, smoking status, depression, chronic pain, need for information, neuroticism, number of previous surgeries, surgery type, perceived physician competence and warmth, and premedication) might moderate the effects of a personalized intervention on preoperative anxiety. Given the exploratory nature of these analyses, the results should be considered hypothesis-generating and may help identify patient characteristics warranting further investigation in adequately powered studies.

## Method

This paper is based on a single-center, prospective, randomized, controlled trial at Marburg University Hospital Department of Anesthesia and Intensive Care. Data collection lasted from March 1, 2021, until January 30, 2023. The main results regarding the intervention effects have been published recently^5^. The present study investigates the moderating role of selected patient characteristics on intervention outcomes.

The study protocol was approved by the Ethics Committee of the University Hospital Marburg, Marburg, Germany (approval No. AZ 148/20). All methods were performed in accordance with the relevant guidelines and regulations and with the Declaration of Helsinki. The trial was prospectively registered in the German Clinical Trials Register (DRKS; registration number: DRKS00024416; date of first registration: 16/02/2021; direct link: https://drks.de/search/en/trial/DRKS00024416).

Procedure, Inclusion and exclusion criteria and informed consent.

The study included (1) adult inpatients (2) scheduled for elective procedures under general anesthesia the following day, (3) who were admitted by the afternoon before surgery and (4) who reported anxiety and a desire for supportive information to deal with their preoperative anxiety. Exclusion criteria included procedures suitable for regional anesthesia alone, illiteracy, language barriers, and visual impairments.

Patients meeting the eligibility criteria 1–3 were invited to complete an anonymous, voluntary initial survey (questionnaire 1), to identify those patients who met inclusion criterion 4. Completing this survey was taken as implied consent. Patients who met all four inclusion criteria and provided both verbal and written informed consent completed a second anxiety assessment (Questionnaire 2) prior to random allocation to either the intervention or control group using a sealed-envelope procedure. Participants in the intervention group selected their preferred form of support—conversation, video, or both—which was then provided by an anesthetist immediately after randomization. The conversation intervention was delivered by anesthetists trained in basic psychotherapeutic techniques, including empathetic listening and addressing patient fears. These discussions focused on individual concerns, using standardized responses to common anxieties to ensure consistency. The video intervention provided a 10-minute overview of the final preoperative process, from the patient’s arrival in the operating area to the induction of anesthesia, with the option to pause for questions.

All participants completed a series of follow-up questionnaires (3 to 7) at predefined intervals to track changes in anxiety. Questionnaire 3 was filled out shortly after the intervention or approximately 45 min after randomization in the control group. Additional questionnaires were completed at standardized times: before bedtime (questionnaire 4), the following morning (questionnaire 5), in the anesthesia induction room (questionnaire 6), and the day after surgery (questionnaire 7). Further details about the questionnaires and study procedures are available in the supplementary materials and the previously published source^5^.

### Assessments

Anxiety was assessed using the validated German version^7^ of the Amsterdam Properative Anxiety and Information Scale (APAIS)^8^, a six-item tool measuring anesthesia-related anxiety (primary outcome), surgery-related anxiety, and information needs. As a secondary measure and internal control, a 0–10 numeric rating scale was also used to evaluate overall, anesthesia-related, and surgery-related anxiety. Total anxiety scores were derived from both tools by summing up surgery- and anesthesia-related anxiety^9^.

We additionally assessed patients’ age (years), (biological) sex, education (in years), existing depression (no vs. yes), current smoking status (no vs. yes), the number of previous surgeries (none vs. 1 or 2 vs. >2), surgery type (minor vs. medium vs. major), and whether patients received anxiolytic premedication (no vs. yes). Patients also rated the anesthetist who conducted the preanesthetic consultation on two single-item numeric rating scales ranging from 0 (“not at all”) to 10 (“very”), assessing perceived physician warmth and perceived physician competence. We further assessed patients’ neuroticism levels using the two respective items of the Big Five Inventory-10^10^. Chronic pain was assessed using a single self-report item (yes/no). No operational definition of chronic pain was provided, and no additional information regarding pain duration, location, intensity, underlying diagnosis, treatment status, whether the planned surgery was related to the painful condition, or concomitant medication use was collected. All of these variables, including patients’ need for information but excluding age and sex (these two variables have been examined as potential moderators of the intervention effects in the previous paper^5^, were examined as potential moderators. The selection of moderator variables was based on the prespecified variables described in the study protocol and the registered statistical analysis plan.

### Statistical analyses

To test whether the above-mentioned variables moderated the intervention effects, we calculated constrained linear mixed models^11,12^ with baseline adjustment, in which the first assessment point (Questionnaire 1) was constrained to be equal between the randomized groups. Models were estimated using maximum likelihood, which uses all available repeated outcome data under the assumption that data are missing at random (MAR). We included a random intercept to account for subject-specific effects, while fixed effects were calculated for the interaction between intervention group by time by moderator and lower order terms. This was done for each potential moderator and all anxiety outcomes separately. In case of significant three-way-interactions, post-hoc tests were used to examine at which assessment time point groups differed statistically significantly. Although the 0–10 Numeric Rating Scale is formally ordinal, it was analyzed as an approximately continuous outcome, consistent with common practice for 11-point rating scales in linear mixed-model analyses. Analyses were conducted using SPSS Statistics software version 29.0 (IBM SPSS Inc., USA). Given the exploratory nature of this secondary analysis, all moderator analyses should be considered hypothesis-generating. To account for multiple testing, p-values were additionally adjusted using the Holm-Bonferroni procedure.

## Results

A total of 122 patients were randomized to the intervention group (*n* = 61) or the control group (*n* = 61). Baseline characteristics have been reported previously^5^ and are reproduced in Table 1 for completeness. The participants had a mean age of 57.4 years (SD = 13.4), and 63.9% (*n* = 78) were female. Participants reported an average of 10.9 years of education (SD = 2.2). Fewer than 10% indicated the scheduled surgery would be their first, while almost one-third reported one or two previous surgeries, and nearly two-thirds had undergone more than two.

Approximately a quarter of participants (*n* = 32) were current smokers, and a similar proportion (*n* = 31) reported chronic pain. Additionally, 17.2% (*n* = 21) reported symptoms of depression. Mean neuroticism scores were 6.9 (SD = 2.0). The mean need for information was 3.07 (SD = 1.1) regarding anesthesia, 3.4 (SD = 1.1) for surgery, and 6.54 (SD = 2.0) for both combined.

**Table 1:** Patient characteristics.

	All patients(*n* = 122)	Control(*n* = 61)	Intervention(*n* = 61)
Age (yr)	57.4 (13.4)	55.8 (13.3)	58.9 (13.3)
Female	78 (63.9)	40 (65.6)	38 (62.3)
Education (yr)			
Missing data = 6	10.9 (2.2)	10.9 (2.5)	10.9 (1.9)
Number of previous surgeries			
None	12 (9.8)	6 (9.8)	6 (9.8)
1–2	37 (30.3)	22 (36.1)	15 (24.6)
> 2	73 (59.8)	33 (54.1)	40 (65.6)
Surgical discipline			
General	41 (33.6)	22 (36.1)	19 (31.1)
Gynecological	30 (24.6)	17 (27.9)	13 (21.7)
Trauma	14 (11.5)	6 (9.8)	8 (13.1)
Dermatological	9 (7.4)	4 (6.6)	5 (8.2)
Oral and maxillofacial	9 (7.4)	4 (6.6)	5 (8.2)
Orthopedic	6 (4.9)	2 (3.3)	4 (6.6)
Urological	6 (4.9)	3 (4.9)	3 (4.9)
Ears, nose and throat	4 (3.3)	3 (4.9)	1 (1.6)
Ophthalmic	2 (1.6)	0	2 (3.3)
Missing data = 1			
Current smoking status			
No	85 (69.7)	38 (62.3)	47 (77.0)
Yes	32 (26.2)	20 (32.8)	12 (19.7)
Missing data = 5			
Chronic pain			
No	89 (73.0)	43 (70.5)	46 (75.4)
Yes	30 (24.6)	16 (26.2)	14 (23.0)
Missing data = 3			
Depression			
No	100 (82.0)	51 (83.6)	49 (80.3)
Yes	21 (17.2)	9 (14.8)	12 (19.7)
Missing data = 1			
Neuroticism			
Missing data = 3	6.9 (2.0)	6.9 (2.0)	6.9 (1.9)
Perceived physician competence			
Missing data = 1	8.48 (1.8)	8.59 (1.9)	8.37 (1.7)
Perceived physician warmth			
Missing data = 1	8.62 (1.7)	8.64 (1.9)	8.60 (1.4)
Premedication			
No	66 (54.1)	40 (65.6)	26 (42.6)
Yes	44 (36.1)	17 (27.9)	27 (44.3)
Missing data = 12			

Data are presented as mean (SD) or n (%). Percentages are based on the total number of participants within each treatment group. Baseline characteristics shown in this table have been previously published in^5^ and are reproduced here for completeness.

Table 2 summarizes the tests of the intervention group × assessment time × moderator interaction terms obtained from the constrained linear mixed models.

**Table 2:** Results of exploratory three-way interaction analyses.

Outcome	Moderator	Three-way interaction (F statistic)
APAIS: Anesthesia-related anxiety	Education (*n* = 116)	*F*(5, 88.649) = 1.047; *p* = .395
	Need for information (*n* = 122)	*F(5, 104.789) = 1.517, p = .191*
	Chronic pain (*n* = 119)	***F***(5,** 102.070) = 2.840**, **p** **= .019**
	Depression (*n* = 121)	*F(5, 111.407) = 1.681, p = .145*
	Smoking (*n* = 117)	*F(5, 95.067) = 0.518, p = .762*
	Neuroticism (*n* = 119)	*F*(5, 90.842) = 1.058, *p* = .389
	Number of previous surgeries (*n* = 122)	*F(10, 97.959) = 0.216, p = .994*
	Surgery type (*n* = 121)	*F(10, 88.880) = 0.511, p = .878*
	Perceived physician competence (*n* = 121)	*F(5, 86.954) = 2.276, p = .054*
	Perceived physician warmth (*n* = 121)	*F(5, 98.395) = 0.629, p = .678*
	Premedication (*n* = 110)	*F(5, 104.830) = 1.191, p = .318*
APAIS: Surgery-related anxiety	Education (*n* = 116)	*F(5, 107.646) = 1.211, p = .304*
	Need for information (*n* = 122)	*F(5, 114.988) = 0.314, p = .904*
	Chronic pain (*n* = 119)	*F(5, 108.547) = 1.384, p = .236*
	Depression (*n* = 121)	*F(5, 113.917) = 1.142, p = .342*
	Smoking (*n* = 117)	*F(5, 109.391) = 0.727, p = .605*
	Neuroticism (*n* = 119)	*F(5, 109.763) = 1.139, p = .344*
	Number of previous surgeries (*n* = 122)	*F(10, 111.187) = 1.142, p = .338*
	Surgery type (*n* = 121)	*F(10, 109.545) = 0.405, p = .942*
	Perceived physician competence (*n* = 121)	*F(5, 112.162) = 0.381, p = .861*
	Perceived physician warmth (*n* = 121)	*F(5, 112.306) = 0.172, p = .973*
	Premedication (*n* = 110)	*F(5, 102.879) = 0.517, p = .763*
APAIS: Total anxiety (sum)*	Education (*n* = 116)	*F(5, 110.745) = 1.240, p = .295*
	Need for information (*n* = 122)	*F(5, 120.494) = 1.174, p = .326*
	Chronic pain (*n* = 119)	*F(5, 112.716) = 1.626, p = .159*
	Depression (*n* = 121)	*F(5, 119.283) = 2.149, p = .064*
	Smoking (*n* = 117)	*F(5, 112.569) = 0.760, p = .580*
	Neuroticism (*n* = 119)	*F*(5, 113.099) = 1.065, *p* = .383
	Number of previous surgeries (*n* = 122)	*F(10, 114.900) = 0.530, p = .866*
	Surgery type (*n* = 121)	*F(10, 113.771) = 0.535, p = .862*
	Perceived physician competence (*n* = 121)	*F(5, 114.518) = 1.032, p = .402*
	Perceived physician warmth (*n* = 121)	*F(5, 114.956) = 0.301, p = .911*
	Premedication (*n* = 110)	*F(5, 107.416) = 1.095, p = .367*
NRS: Anesthesia-related anxiety	Education (*n* = 116)	*F(5, 113.651) = 1.247, p = .292*
	Need for information (*n* = 122)	*F(5, 122.726) = 0.377, p = .863*
	Chronic pain (*n* = 119)	***F***(5,** 115.517) = 2.359**, **p** **= .044**
	Depression (*n* = 121)	*F(5, 123.823) = 1.201, p = .313*
	Smoking (*n* = 117)	*F(5, 116.730) = 1.286, p = .275*
	Neuroticism (*n* = 119)	*F(5, 116.218) = 0.287, p = .920*
	Number of previous surgeries (*n* = 122)	*F(10, 117.865) = 0.399, p = .945*
	Surgery type (*n* = 121)	*F(10, 117.234) = 0.931, p = .507*
	Perceived physician competence (*n* = 121)	*F(5, 117.836) = 0.895, p = .487*
	Perceived physician warmth (*n* = 121)	*F(5, 117.356) = 1.011, p = .414*
	Premedication (*n* = 110)	*F(5, 109.546) = 0.943, p = .456*
NRS: Surgery-related anxiety	Education (*n* = 116)	*F(5, 113.790) = 0.247, p = .941*
	Need for information (*n* = 122)	*F(5, 122.272) = 0.735, p = .598*
	Chronic pain (*n* = 119)	*F(5, 116.611) = 1.327, p = .258*
	Depression (*n* = 121)	*F(5, 123.230) = 0.394, p = .852*
	Smoking (*n* = 117)	*F(5, 117.486) = 0.927, p = .466*
	Neuroticism (*n* = 119)	*F(5, 116.308) = 0.997, p = .423*
	Number of previous surgeries (*n* = 122)	*F(10, 118.652) = 0.651, p = .767*
	Surgery type (*n* = 121)	*F(10, 117.747) = 0.724, p = .701*
	Perceived physician competence (*n* = 121)	*F(5, 118.807) = 0.337, p = .889*
	Perceived physician warmth (*n* = 121)	*F(5, 118.445) = 0.669, p = .648*
	Premedication (*n* = 110)	*F(5, 109.575) = 0.585, p = .711*
NRS: Total anxiety (sum)*	Education (*n* = 116)	*F(5, 114.374) = 0.511, p = .767*
	Need for information (*n* = 122)	*F(5, 122.191) = 0.453, p = .810*
	Chronic pain (*n* = 119)	*F(5, 116.287) = 1.560, p = .177*
	Depression (*n* = 121)	*F(5, 122.414) = 0.921, p = .470*
	Smoking (*n* = 117)	*F(5, 117.608) = 1.199, p = .314*
	Neuroticism (*n* = 119)	*F(5, 116.910) = 0.491, p = .782*
	Number of previous surgeries (*n* = 122)	*F(10, 118.969) = 0.536, p = .862*
	Surgery type (*n* = 121)	*F(10, 117.883) = 0.904, p = .532*
	Perceived physician competence (*n* = 121)	*F(5, 118.614) = 0.527, p = .756*
	Perceived physician warmth (*n* = 121)	*F(5, 118.376) = 0.831, p = .530*
	Premedication (*n* = 110)	*F(5, 110.313) = 0.534, p = .750*
NRS: Overall anxiety	Education (*n* = 114)	*F(5, 114.321) = 1.006, p = .418*
	Need for information (*n* = 119)	*F(5, 121.612) = 1.147, p = .340*
	Chronic pain (*n* = 119)	*F(5, 114.927) = 1.208, p = .310*
	Depression (*n* = 118)	*F(5, 122.367) = 0.878, p = .498*
	Smoking (*n* = 114)	*F(5, 114.306) = 1.563, p = .176*
	Neuroticism (*n* = 116)	*F*(5, 114.742) *= *0.855*, p =* .514
	Number of previous surgeries (*n* = 119)	*F(10, 118.927) = 0.723, p = .701*
	Surgery type (*n* = 119)	*F(10, 116.769) = 0.883, p = .551*
	Perceived physician competence (*n* = 118)	*F*(5, 116.409)* = 0.409, p =* .842
	Perceived physician warmth (*n* = 118)	*F*(5, 116.347) *= 0.629, p =* .678
	Premedication (*n* = 108)	*F*(5, 108.561) *=* 0.455*, p =* .809

APAIS, Amsterdam Preoperative Anxiety and Information Scale. NRS, Numeric Rating Scale. P-values are unadjusted. After Holm-Bonferroni correction across all 77 moderator tests, none of the effects remained statistically significant. * Total anxiety (sum) is the sum of anesthesia- and surgery-related anxiety.

None of the examined moderator effects remained statistically significant after Holm-Bonferroni correction across the 77 exploratory interaction tests. The smallest unadjusted p-values were observed for chronic pain with regard to anesthesia-related anxiety assessed by the APAIS (*p* = .019) and the corresponding Numeric Rating Scale (*p* = .044). Because both instruments assess the same construct, these observations should be interpreted as complementary exploratory findings rather than independent evidence of a moderator effect. The smallest unadjusted p-value (*p* = .019) exceeded the first Holm-Bonferroni threshold of α = 0.000649 (0.05/77).

**Fig. 1:**
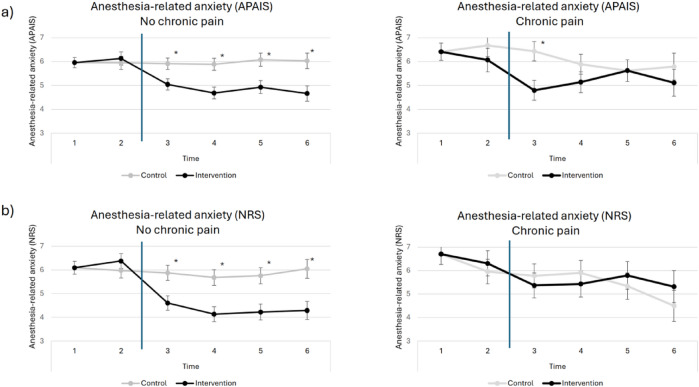
Exploratory trajectories of anesthesia-related anxiety according to intervention group and chronic pain status for the APAIS (**a**) and NRS (**b**). The left panels show patients without chronic pain, and the right panels show patients with chronic pain. The numbers on the x-axis represent the assessment time points in relation to randomization (vertical line): 1, in the afternoon after preanesthetic consultation; 2, in the afternoon immediately before randomization; 3, shortly after the intervention (intervention group) or approximately 45 min after randomization (control group); 4, before bedtime; 5, after waking up the following morning; and 6, after arrival in the anesthesia induction room. APAIS, Amsterdam Preoperative Anxiety and Information Scale; NRS, Numeric Rating Scale. Because chronic pain yielded the smallest unadjusted p-values among all examined moderators, these trajectories are shown for descriptive purposes. The corresponding moderator effects did not remain statistically significant after Holm-Bonferroni correction for multiple testing.

Figure 1 descriptively illustrates the observed trajectories of anesthesia-related anxiety according to chronic pain status. Chronic pain is shown because it yielded the smallest unadjusted p-values among the examined exploratory moderator analyses; however, these findings did not remain statistically significant after correction for multiple testing.

## Discussion and conclusion

### Discussion

This exploratory secondary analysis found little evidence that the effects of the personalized preoperative anxiety intervention varied systematically according to the examined sociodemographic, psychological, or clinical patient characteristics. After adjustment for multiple testing, none of the examined moderator effects remained statistically significant. Therefore, the present findings do not provide confirmatory evidence for treatment effect heterogeneity across the investigated patient characteristics.

The intervention examined in this study has previously been shown to produce statistically significant and clinically meaningful reductions in preoperative anxiety among patients seeking support [5]. The present exploratory analyses provided no confirmatory evidence that these effects differed according to the examined patient characteristics. However, it is important to note that the present study was powered to detect treatment effects, but not moderator effects. Detecting interaction effects generally requires substantially larger sample sizes than detecting main effects. Consequently, the absence of statistically significant moderator findings should not be interpreted as evidence that intervention effects are identical across all patient subgroups.

Among the examined moderators, chronic pain yielded the smallest unadjusted p-values with regard to anesthesia-related anxiety outcomes as assessed by two complementary measures of the same construct. However, these findings did not remain statistically significant after adjustment for multiple testing and were based on a relatively small subgroup of patients with chronic pain (*n* = 31; intervention: *n* = 15; control: *n* = 16). Consequently, these observations should be considered exploratory and hypothesis-generating. Nevertheless, the observation that the strongest unadjusted signals were found for chronic pain may warrant further investigation in adequately powered confirmatory studies. In addition, because participants in the intervention group were allowed to select their preferred intervention modality (conversation, video, or both), differential modality selection cannot be excluded as an alternative explanation for the observed exploratory findings regarding chronic pain.

Although chronic pain has previously been associated with elevated psychological distress, fear-related cognitions, and reduced treatment expectations [13–15], the present study does not allow conclusions regarding the mechanisms underlying the observed exploratory findings. In particular, the current analyses did not examine whether patients with chronic pain differed in baseline anxiety or other characteristics that might explain the observed pattern. Furthermore, chronic pain was assessed using a single self-report item without information on pain duration, location, severity, underlying diagnosis, treatment status, whether the planned surgery was related to the painful condition, or concomitant analgesic and psychotropic medication use. Consequently, alternative explanations for the observed findings—including differences in baseline anxiety severity, medication use, or intervention modality selection—cannot be excluded. Future adequately powered confirmatory studies incorporating a more comprehensive assessment of chronic pain characteristics and associated treatments are needed to determine whether chronic pain truly moderates intervention effects.

### Conclusion

In this exploratory secondary analysis, little evidence was found that the effects of a personalized intervention to reduce preoperative anxiety varied systematically according to the examined patient characteristics. Although chronic pain yielded the strongest indication of potential effect modification, this finding did not remain statistically significant after correction for multiple testing and should therefore be considered hypothesis-generating. Future adequately powered studies are needed to determine whether specific patient characteristics influence response to personalized preoperative anxiety interventions.

## Supplementary Information

Below is the link to the electronic supplementary material.

Supplementary Material 1

## Data Availability

The datasets used and/or analyzed during the current study are available from the corresponding author upon reasonable request.
