# Supplementary material for: Exploratory analyses of potential moderators of a personalized intervention to reduce preoperative anxiety: a secondary analysis of a randomized clinical trial
Source: Sci Rep. 2026 Jul 22;16:22942. doi: 10.1038/s41598-026-63192-w (PMC13392235; doi:10.1038/s41598-026-63192-w)
Supplement: Supplementary file 1 — Supplementary Material 1 [file 41598_2026_63192_MOESM1_ESM.docx]

**Supplementary Material - Questionnaire:**

Part 1) Amsterdam Preoperative Anxiety and Information Scale (APAIS)

1.1 English version of the APAIS as presented by Moerman and colleagues^1^


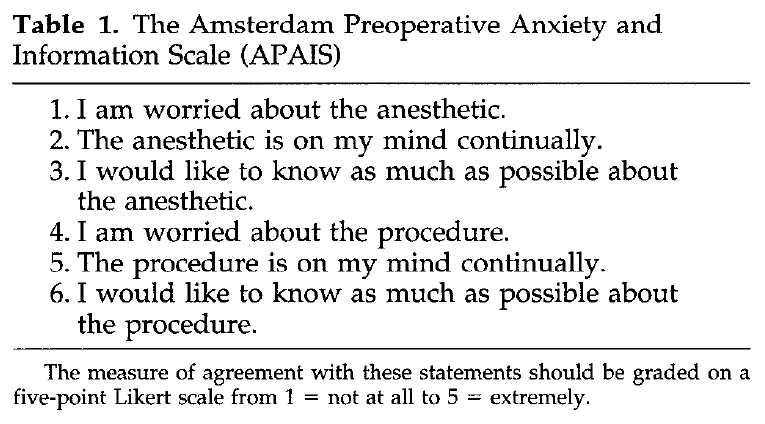


1.2 German version of the APAIS, adapted from Berth and colleagues^2^

|  | **1**  (gar nicht) | **2**  (wenig) | **3**  (mittel) | **4**  (stark) | **5**  (extrem) |
| --- | --- | --- | --- | --- | --- |
| 1. Ich mache mir Sorgen über die Anästhesie (Narkose) |  |  |  |  |  |
| 2. Die Anästhesie (Narkose) geht mir ständig durch den Kopf |  |  |  |  |  |
| 3. Ich möchte so viel wie möglich über die Anästhesie (Narkose) wissen |  |  |  |  |  |
| 4. Ich mache mir Sorgen über die Operation |  |  |  |  |  |
| 5. Die Operation geht mir ständig durch den Kopf |  |  |  |  |  |
| 6. Ich möchte so viel wie möglich über die Operation wissen |  |  |  |  |  |

1.3 Description of the APAIS adapted from Eberhart and colleagues^3^

The APAIS contains six items (statements) with a score range from 1-5. It includes two items to assess the magnitude of patient’s anxiety about anesthesia (APAIS-An-A, items 1 +2; sum score range 2-10), two items concerning anxiety about surgery (APAIS-Su-A, items 4 + 5; sum score range 2-10) and two items to assess patient’s need for information (APAIS-I, items 3+6; sum score range 2-10) with higher scores indicating a higher level of anxiety / need for information. Total preoperative anxiety (APAIS-T-A, items 1, 2, 4 +5; sum score 4–20) is the sum of anesthesia- and surgery-related anxiety (APAIS-An-A plus APAIS-Su-A). The APAIS was validated in many countries using different languages (e.g. ^4-6^). Recently published results of a large survey demonstrated that the reliability (Cronbach’s α) of the four anxiety items (“anxiety scale”) and of the two information items (“information scale”) were 0.87 and 0.74, respectively.^7^
We calculated reliability for the two items measuring anesthesia-related anxiety (Spearman-Brown coefficient: 0.904), and for the two items assessing surgery-related anxiety (Spearman-Brown coefficient: 0.88). The results indicate good reliability and are consistent with the previously reported reliability of the APAIS total anxiety score (Cronbach’s α 0.86 [Moerman et al. 1996 – ref.24]).
For assessing the validity, we analyzed correlations for the sum score of the anesthesia-related anxiety using the APAIS and the NRS assessment for anesthesia-related anxiety (r=.719, p<.001) as well as for the sum score of the surgery-related anxiety using the APAIS and the NRS assessment for surgery-related anxiety (r=.782, p<.001). The results indicate good validity.

Part 2) Description of questionnaires 1-7

Questionnaire 1 is a modified version of the questionnaire used and described in detail previously.^8^ It consists of four sections:
Section 1 asked for age, gender, education, variables related to the scheduled surgery, previous surgeries, smoking, chronic pain, depression, and neuroticism.
In section 2 patients were asked whether they were anxious about the planned surgery and / or the anesthesia required for it (no/yes). Those who answered “yes” were asked (a) whether the reported anxiety was related to surgery, anesthesia or both, (b) what exactly they were scared of using free text answers, (c) whether the anxiety was perceived as emotionally distressful or as unsettling (no/yes) and how strong the emotional distress was using an NRS ranging from 0 (no distress) to 10 (extreme distress), and (d) whether they would welcome to get anesthesiologists’ support in coping with their anxiety (no/yes). Patients who reported a desire for support (yes) were asked whether (e) they would welcome support in coping with their anxiety by means of getting additional information. If that was the case patients were asked to choose the option that would help the most: (e-1) additional information provided by a video with the option of asking questions to the physician performing the support measure, (e2) additional information provided during an empathic conversation with the physician performing the support measure, (e-3) both video and conversation. Next, patients were asked to outline in key words what kind of support would be best for them if none of the offered support measures (e1-3) would be an option.
Section 3 contained three NRS ranging from 0 (no anxiety) to 10 (extreme anxiety) to explore patients’ intensity of anxiety related to anesthesia, to surgery and intensity of overall anxiety.
Section 4 comprised the validated German version ^2^ of the APAIS ^1^. It has been described in extenso in numerous publications (e.g. ^7^) and validated versions exist in many languages (e.g. languages (e.g. ^4-6^).

Questionnaire 2 included two sections:
Section 1 is identical with section 3 of questionnaire 1 (NRS anxiety)
Section 2 is identical with section 4 of questionnaire 1 (APAIS)

Questionnaires 3-6 consisted of three sections:
Section 1 is identical with section 3 of questionnaire 1 (NRS anxiety)
Section 2 is identical with section 4 of questionnaire 1 (APAIS)
Section 3 comprised 3 items asking patients to compare their present anxiety level about anesthesia, surgery and overall anxiety to the corresponding anxiety level prior to randomization, i.e. the time before subjects completed questionnaire 2. Patients could choose between the following options: the anxiety is a) markedly weaker, b) a little weaker, c) the same, d) a little stronger, e) markedly stronger.

Questionnaire 7 for patients in the control group only had one question concerning the overall satisfaction with medical care since admission to hospital using an NRS ranging from 0 (not satisfied at all) to 10 (very satisfied). In addition, patients of the intervention group were asked a) whether the support measure was helpful to cope with their anxiety (no/yes), b) concerning the degree of the support using an NRS ranging from 0 (none) to 10 (extreme), and c) whether they would recommend the chosen support measure to a friend in case they would undergo a similar procedure (no/yes).
